# Hexaconazole-Micelle Nanodelivery System Prepared Using Different Surfactants for Ganoderma Antifungal Application

**DOI:** 10.3390/molecules26195837

**Published:** 2021-09-26

**Authors:** Isshadiba Faikah Mustafa, Mohd Zobir Hussein, Abu Seman Idris, Nur Hailini Zainol Hilmi, Sharida Fakurazi

**Affiliations:** 1 Materials Synthesis and Characterization Laboratory, Institute of Advanced Technology, Universiti Putra Malaysia, Serdang 43400, Selangor, Malaysia; isshyka1202@gmail.com; 2 Malaysian Palm Oil Board (MPOB), No. 6, Persiaran Institusi, Bandar Baru Bangi, Kajang 43000, Selangor, Malaysia; idris@mpob.gov.my (A.S.I.); hailini@mpob.gov.my (N.H.Z.H.); 3 Department of Human Anatomy, Faculty of Medicine and Health Sciences, Universiti Putra Malaysia, Serdang 43400, Selangor, Malaysia; sharida@upm.edu.my

**Keywords:** hexaconazole, surfactants, micelle, antifungal, nanodelivery, stability

## Abstract

Reports on fungicide-based agronanochemicals in combating disastrous basal stem rot disease in the oil palm industry are scant. Herein, we describe the potential of fungicide nanodelivery agents based on hexaconazole-micelle systems produced using three different surfactants; sodium dodecylbenze sulfonate (SDBS), sodium dodecyl sulfate (SDS) and Tween 80 (T80). The resulting nanodelivery systems were characterized and the results supported the encapsulation of the fungicide into the micelles of the surfactants. We have investigated in detail the size-dependent effects of the as-synthesized micelles towards the inhibition growth of *Ganoderma Boninense* fungi. All the nanodelivery systems indicate that their size decreased as the surfactant concentration was increased, and it directly affects the fungal inhibition. It was also found that Tween 80, a non-ionic surfactant gave the lowest effective concentration, the EC_50_ value of 2, on the pathogenic fungus *Ganoderma boninense* compared to the other anionic surfactants; SDBS and SDS. This study opens up a new generation of agronanofungicide of better efficacy for Ganoderma disease treatment.

## 1. Introduction

Basal stem rot disease (BSR) is a leading concern in the palm oil upstream industry. This soil-borne disease is caused by a facultative saprophyte organism called *Ganoderma boninense*. This fungus has contributed to major economic loss to countries in Southeast Asia, especially Malaysia. Some control strategies have been introduced but the use of currently available fungicide resulted in less efficiency, user and environmental concerns. The application of hexaconazole fungicide in BSR management has been introduced earlier and its ability to lengthen the lifespan of infected oil palm was proven [1]. However, the direct application of hexaconazole as a fungicide in battling BSR disease is not reaching the maximal effect towards the target site due to leaching and dissipation problems [2,3,4]. Therefore, many formulations have been developed to improve and increase the efficiency of current strategies. The nanotechnology approach in developing new formulations is progressing tremendously. Previously works have documented nanoformulations for handling BSR disease using hexaconazole-based fungicide on different delivery systems such as layered double hydroxide (LDH) [5] and chitosan [6] and the results are very promising. The use of surfactants in nanoformulations with their superior hydrophobicity–hydrophilicity and antagonistic properties towards pathogenic microbes [7] has been very advantageous. The surfactants have been shown to play a promising role in formulating agronanochemical products as they can reduce surface tension, maintain the turgidity of droplets on the target site and lower the pH solution to ensuring the ingredients to be preserved. The effect of different surfactants towards fungal disease in plants such as *Fusarium* head blight, *Beauveria bassiana* [8] and powdery mildew [9] have been reported recently. Currently, the study on the use of surfactants for the treatment of the *Ganoderma boninense* pathogen, a causal pathogen for BSR disease, is still lacking. Here, we describe our work on the design and synthesis of micelle-based hexaconazole nanodelivery systems using three different surfactants; sodium dodecylbenzene sulfonate (SDBS), sodium dodecyl sulfate (SDS) and Tween 80 (T80). They were used separately, acted as the shell and the fungicide, hexaconazole was encapsulated into it, acted as the core. Their physicochemical and fungicidal potency towards *Ganoderma boninense* pathogen were characterized and compared.

## 2. Results and Discussion

### 2.1. Critical Micelle Concentration

**Figure 1:**
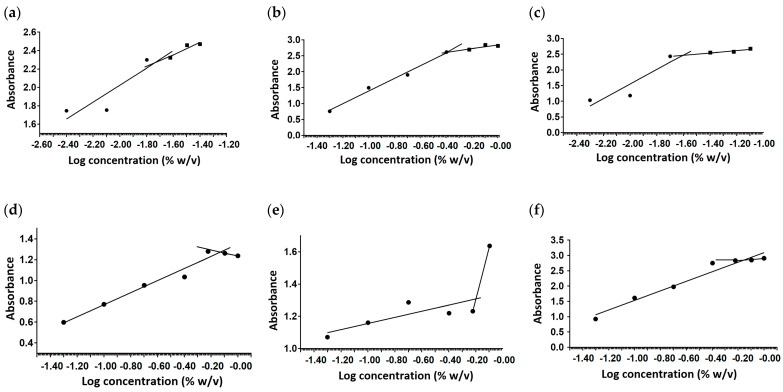
CMC diagram of SDBS (**a**), SDS (**b**), T80 (**c**), HMBS (**d**), HMDS (**e**), and HMT (**f**) using the pyrene fluorescence probe.

The hexaconazole micelles nanodelivery systems based on three surfactants; HMBS, HMDS and HMT were prepared by a simple method, the nanoprecipitation method. The loading content of hexaconazole for HMBS, HMDS and HMT is 14.9%, 35.7%, 19.1% with an encapsulation efficiency of 40.7%, 52.1% and 35.4%, respectively. The formation of three hexaconazole micelles was supported by the CMC determination as shown in Figure 1. The CMC value of HMBS, HMDS, and HMT was found to be 0.72, 0.63 and 0.67% *w*/*v*, respectively. It was observed that after the incorporation of hexaconazole in the surfactant solution, the value of CMC increases slightly compared to bare surfactants of SDBS, SDS and Tween 80, with a value of 0.01, 0.39 and 0.02% *w*/*v*. The slight changes of CMC value in micelle formation showing the CMC synergism. The presence of an organic solvent in the mixture also affects the enthalpy and entropy values in the micellization process, thus influencing the ordered structure through hydrogen bonding in an aqueous solution [10]. The finding indicates that the presence of surfactants in the mixture increases the mixture’s hydrophilicity, solubility and CMC.

### 2.2. Powder X-ray Diffraction

**Figure 2:**
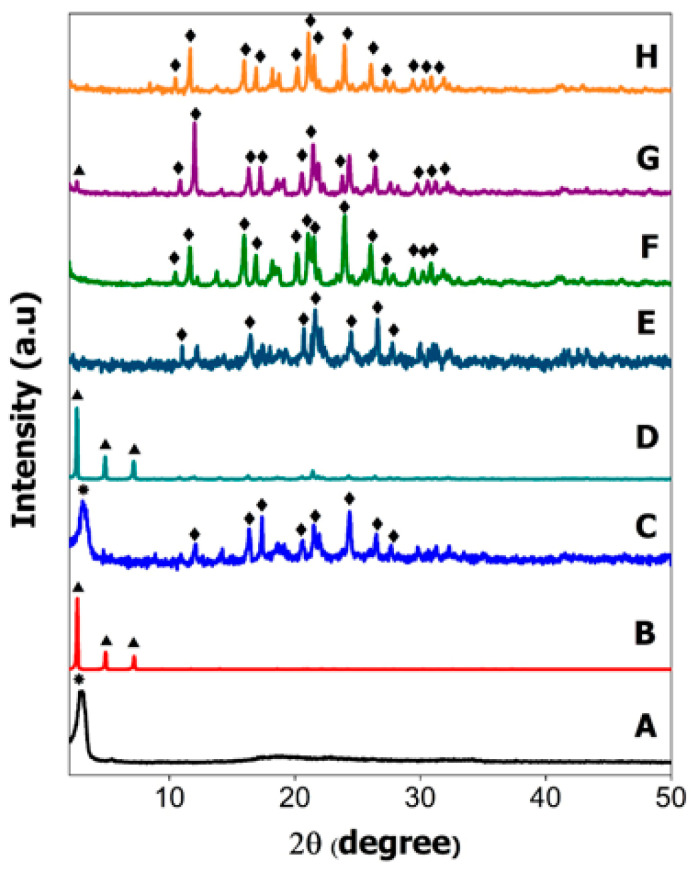
XRD patterns of pure SDBS (**A**), SDS (**B**), physical mixture of HMBS (**C**), HMDS (**D**), HMT (**E**), and their hexaconazole-micelle nanodelivery systems; HMBS (**F**), HMDS (**G**) and HMT (**H**).

Powder X-Ray Diffraction (XRD) analysis was conducted for all the hexaconazole-micelles delivery systems prepared using surfactants; sodium dodecylbenzene sulfonate (HMBS), sodium dodecyl sulfate (HMDS) and Tween 80 (HMT) and their physical mixture counterparts (Figure 2).

Based on XRD patterns of the physical mixture, the results obtained are comparable to the peaks shown for pure surfactants (Figure 2A,B). The XRD pattern of HMBS (Figure 2C) shows a broad peak at 3.22° (*) indicating SDBS crystal peak while HMDS (Figure 2D) has exhibited some distinct peaks below 10° such as 2.62, 4.9 and 7.18° (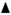) signifying that SDS surfactant also has crystalline property in the mixture. The high intensity of the XRD pattern of SDS in the HMDS sample has suppressed the XRD reflection peak of hexaconazole. Similar reflection peaks at 10.44, 11.64, 15.94, 16.9, 20.24, 21.08, 21.54, 23.94, 26.04, 27.18, 29.4, 30.34 and 30.82° (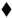) can be seen in the XRD patterns, due to hexaconazole with only some changes in their intensity. Assuming that all the surfactants—SDBS, SDS and T80—used in this work have no reflection peak in their XRD patterns of the as-synthesized micelles (Figure 2F–H), it can be concluded that the surfactants formed the micelle system in an amorphous state [11].

### 2.3. Fourier-Transform Infrared Spectroscopy

**Figure 3:**
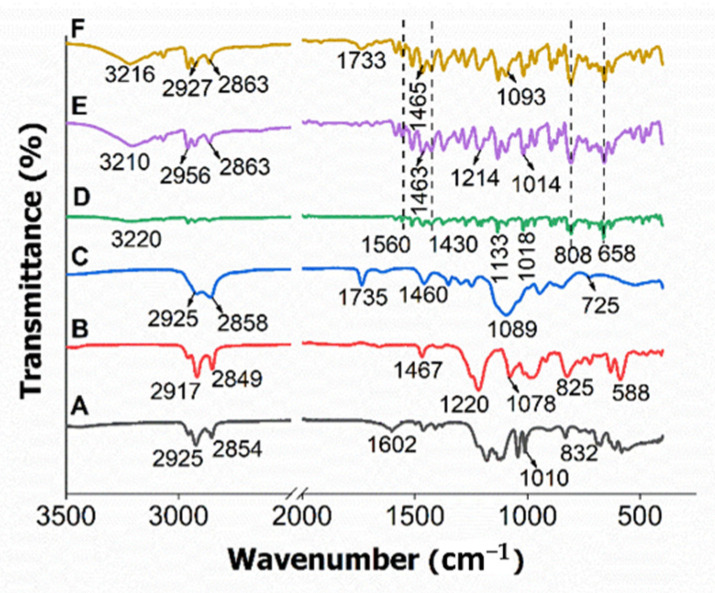
FTIR spectra of the physical mixture of SDBS (**A**), SDS (**B**), T80 (**C**), and their hexaconazole-micelle nanodelivery systems of HMBS (**D**), HMDS (**E**) and HMT (**F**).

The Fourier-transform infrared (FTIR) spectra of HMBS, HMDS and HMT confirmed the encapsulation of hexaconazole into their surfactant micelles systems, as shown in Figure 3. The major FTIR bands of the pure surfactants also have been included, so that a clearer picture of the resulting chemical properties of the resulting hexaconazole-micelles samples can be obtained.

In FTIR spectra of SDBS (Figure 3A), there was symmetric and asymmetric CH_2_/CH_3_ vibration mode at 2925 and 2854 cm^−1^, C=C aromatic at absorption band of 1602 cm^−1^ and C-H bending of benzene at 1010 and 832 cm^−1^ [12] can be observed. For SDS (Figure 3B), the C-H bond vibration was observed at 2917 and 2849 cm^−1^, bands at 1467 cm^−1^ were assigned to CH_2_ stretching and bending mode, the bands at 1220 and 1078 cm^−1^ representing SO_2_ asymmetric in vibration and asymmetric C-H bending of CH_2_ group were detected at 825 and 588 cm^−1^ [13]. In T80 FTIR spectra (Figure 3C), two broad bands of 2925 and 2858 cm^−1^ belonged to C-H stretching of the methylene group and the 1735 cm^−1^ band was due to C=O stretching. The absorption band at 1460 cm^−1^ indicates the C-H stretching of a methylene group, and additional bands at 1089 and 725 cm^−1^ refer to the C-O-C stretching and CH_2_ rocking mode, respectively [14].

After the encapsulation of hexaconazole into the micelle of the surfactant, four characteristic peaks of hexaconazole; C=N, aromatic ring structure, C-H bend and a strong C-Cl stretching band at 1560, 1430, 808 and 658 cm^−1^, respectively, were observed in all the three micelle systems, as shown in Figure 3D–F, respectively. These bands indicate the formation of the nanodelivery system, where hexaconazole was encapsulated into the surfactant-micelles. The first broad band shown by each micelle system was shifted between 3210–3220 cm^−1^, possibly due to the weak electrostatic force of the attachment of ions from the hexaconazole moiety with H atoms of the hydroxyl group.

HMBS spectrum (Figure 3D) also shows the SDBS presence; the C-H bending of benzene is observed at 1133 and 1018 cm^−1^. The band intensity of SDBS at 1184 cm^−1^ is decreased, which may be due to the dissociation of the sulfonate group to forming the acyl group. This formation was observed when the HMBS sample exhibited a sharp band at 1133 cm^−1^ indicating the mixture of hexaconazole and SDBS has generated a bonding between carbonyl and carbon group (acyl group), automatically decreasing the intensity of the alkoxy group at 1043 cm^−1^ where less of the alkyl group was bonded directly to the oxygen atoms.

HMDS shows major bands of SDS surfactant at 2956 and 2863 cm^−1^, assigned to C-H bond vibration, 1463 cm^−1^ for CH_2_ stretching and bending mode and 1214 cm^−1^ related to the SO_2_ asymmetric vibration (Figure 3E). HMT displays C-H stretching of methylene group at 2927 and 2863 cm^−1^, C=O stretching at 1733 cm^−1^, C-H bending of methylene group at 1465 cm^−1^ and C-O-C stretching at 1093 cm^−1^ which are associated with the T80 surfactant (Figure 3F).

### 2.4. Thermal Analysis

**Figure 4:**
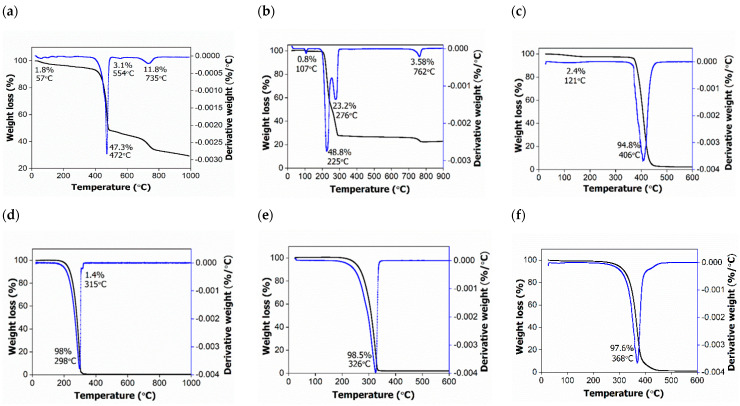
TGA/DTG thermograms of surfactants (**a**) SDBS; (**b**) SDS; (**c**) T80, and their hexaconazole-micelle nanodelivery systems: (**d**) HMBS; (**e**) HMDS; and (**f**) HMT.

Figure 4 depicts the TGA/DTG curves of all the pure surfactants, HMBS, HMDS and HMT samples. SDBS surfactant has shown four stages of weight loss, observed at 57, 472, 554 and 735 °C, with percentage loss of 1.8, 47.3, 3.1 and 11.8%, respectively (Figure 4A). The first weight loss step indicates the removal of adsorbed and structural water molecules. The second one is due to the thermal degradation of the alkyl chain of the molecule, the third and fourth weight loss at around 500 °C may be attributed to the thermal decomposition of the benzene ring, and leaving only surfactant head, forming sodium hydrogen sulfite (NaHSO_3_) [15].

The thermograms of the SDS surfactant (Figure 4B) exhibit four stages of weight loss. The first thermal decomposition which lies at 107 °C with a loss of 0.8% normally comes from water adsorption. The second and third thermal events with 23–49% weight loss are referring to the oxidation of the total alkyl chain of dodecyl sulfate anions that are present in SDS [16]. The last stage, corresponding to 3.58%, may be related to metallic sulfate formation, similar to the SDBS compound.

Based on the results, the occurrence of a few temperature differences in SDS could possibly be caused by incomplete alkyl chain thermal degradation. As an example, the moiety started to degrade after the elimination step of its physically adsorbed water, but it was not fully degraded, therefore it continued to decompose again in the second step until the complete degradation process had occurred [17]. Figure 4C is the thermogram for T80 surfactant, showing two thermal events—a thermal loss at 121 and 406 °C with a value of 2.4 and 94.8%—which correspond to the attached water removal and polyoxyethylene (POE) chains, respectively [18].

The TGA curves of the hexaconazole-micelle nanodelivery system are shown in Figure 4D–F. For all three samples, they show thermal degradation at a single temperature interval between 290–370 °C with a mass loss between 97–98% and were assigned to the degradation of hexaconazole fungicide in the micelles. For HMBS, there is another thermal event at 315 °C with a small loss of 1.4%, which corresponds to the evaporation of volatile compounds such as ionic sulphonates, as indicated by FTIR analysis [19]. In light of all the TGA/DTG results, it can be concluded that the addition of surfactants had increased the thermal stability of the fungicide degradation as was discussed previously [5].

### 2.5. Differential Scanning Calorimetry

**Figure 5:**
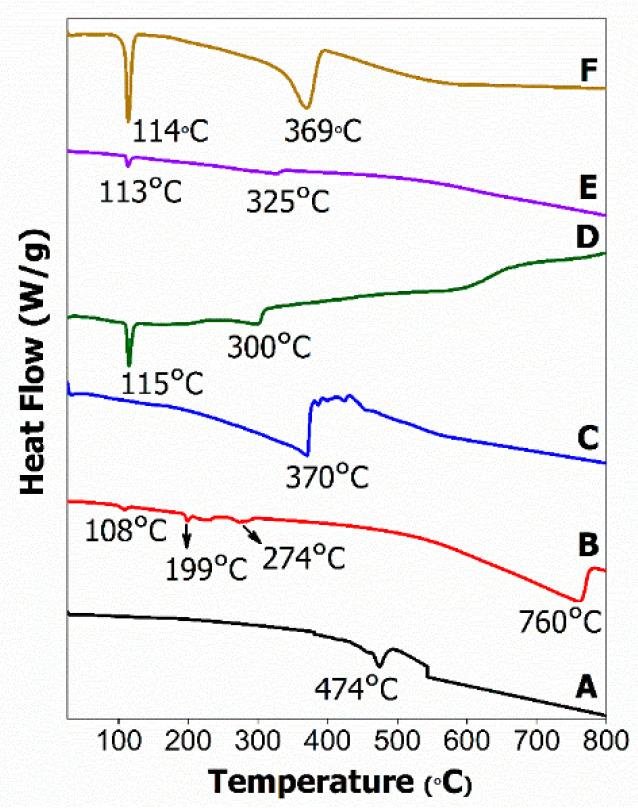
The differential scanning calorimeter thermograms of surfactants, SDBS (**A**); SDS (**B**); T80 (**C**), and their hexaconazole-micelle nanodelivery systems: (**D**) HMBS; (**E**) HMDS; and (**F**) HMT.

The melting activity of pure surfactants and their hexaconazole-micelle nanodelivery systems; HMBS, HMDS and HMT were examined using the differential scanning calorimeter method as shown in Figure 5.

The endothermic peak at 474 °C on the SDBS thermogram (Figure 5A) is referred to as the SDBS melting point followed by decomposition which coincides with weight loss in the TGA curve (Figure 4B) of about 47.3% at 472 °C. For the SDS thermogram (Figure 5B), the occurrence of four endothermic events was observed. The first stage at 108 °C was associated with the detachment of physically and chemically attached water molecules, while the second and third thermal events with 23–49% weight loss at 199 and 274 °C, respectively, referred to the oxidation of total alkyl chain of dodecyl sulfate anions that were present in the SDS moiety. The last stage occurrence at 760 °C, corresponding to 3.58% mass loss, may be related to the oxidation process producing metallic sulfate. The decomposition step in the T80 surfactant (Figure 5C) at 370 °C is highly related to the main group in surfactant, which is polyoxyethylene (POE) chains.

Figure 5D–F show the thermograms of HMBS, HMDS and HMT micelles, showing the same range of endothermic events. All the micelle curves demonstrate two endothermic events, observed at 113–115 °C and 300–370 °C. The first step was associated with strongly held water molecules while the second step was due to the hexaconazole fungicide melting point, which matched with the results of thermal events in its TGA curve for the micelle systems (Figure 4E,F), as discussed earlier. Since no peak can be observed at the surfactant melting point, this infers that all the surfactants were dispersed in the micelle samples.

### 2.6. Surface Morphology

**Figure 6:**
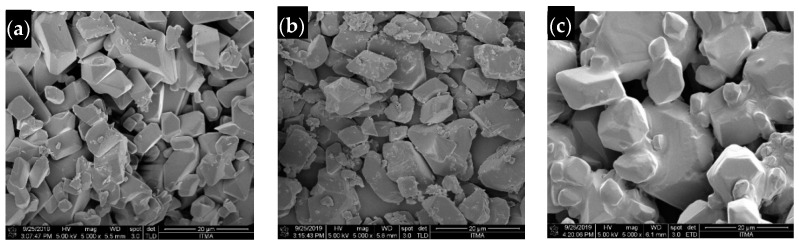
Field emission scanning electron micrographs of (**a**) HMBS, (**b**) HMDS and (**c**) HMT under 5000× magnifications.

Figure 6 illustrates the FESEM micrograph of hexaconazole-micelles nanodelivery formulations. As shown in the figure, all the micelles show irregular nanocube shapes and they are agglomerated together. The latter is due to the drying process of the sample.

### 2.7. Particle Size and Its Stability

Apart from chemical properties, we also explored the effect of surfactant type and its concentration on the size of the resulting hexaconazole-micelle nanodelivery systems. This is to study the size-efficacy relationship of the system towards the inhibition of the *Ganoderma boninense* pathogen. The micelles were also prepared at a higher concentration than the CMC point, to obtain the maximum effect of the surfactants [20]. The micelles with particle sizes below 100 nm were subsequently chosen for this study.

Hexaconazole micelle nanodelivery systems prepared using different concentrations of SDBS (*w*/*w*%) are labeled as HMBS-X, where X indicates the (*w*/*w*%): HMBS-1%, HMBS-2 for 2% and HMBS-4 for 4%, respectively. The same label was also used for hexaconazole–SDS (HMDS) and hexaconazole-T80 (HMT) systems. The resulting micelles systems show a good kinetic micellar growth when the sample achieved a monomodal distribution. The preparation stage that involves sonication and dilution also contributed to the low sample aggregation.

**Figure 7:**
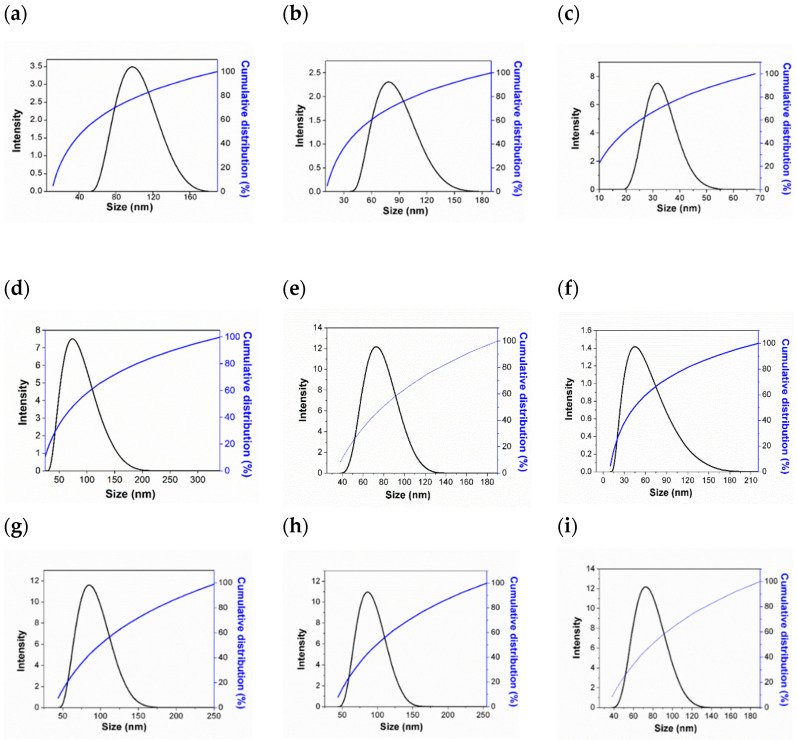
The dynamic light scattering particle size distribution of HMBS-1 (**a**), HMBS-2 (**b**), HMBS-4 (**c**), HMDS-1 (**d**), HMDS-2 (**e**), HMDS-4 (**f**), HMT-6 (**g**), HMT-8 (**h**) and HMT-10 (**i**).

All the prepared samples revealed that the size of the resulting hexaconazole-micelle nanodelivery systems decreased when the concentration of the surfactant added was increased (Figure 7). The densely packed surfactants on the particle surfaces of the hexaconazole caused the surface energy to decrease, resulting in a better homogenous dispersion [21]. The particle size of every system is summarized in Table 1. For SDBS, as the SDS surfactant concentration in the micelle solution was increased from 1% to 4%, the size of the HMBS and HMDS was decreased from 83 nm to 35 nm and from 80 nm to 69 nm, respectively. On the other hand, for the T80 surfactant, the concentration used was 6–10%, resulting in the size of HMT decreased from 88 nm to 74 nm. This trend shows that the particle size is highly dependent on the surfactant concentration and in good agreement with previous works described elsewhere [22]. The particle size obtained by the HMBS system was found to be the lowest, albeit that the surfactant concentration for HMT is higher than that for HMBS and HMDS. Based on this study, therefore, it is suggested that the particle size of the resulting nano micelles is also dependent on the type of the surfactants, as well as its concentration.

After six months of storage, all the nano micelles which were kept in dried form were re-dispersed, and their PSD was measured again. This was undertaken to obtain their final particle size distribution and to see their stability over storage time, such as whether aggregation and coalescence amongst the nano micelles are possible. Based on Table 1, agglomeration or coalesceence between the nanoparticles cannot be ruled out. The changes in PSD might be due to the particle–particle interactions, due to an increase in the surface free energy. The results indicate that the PSD for all the systems was gradually increased to a range of 90–150 nm over the 6 months of storage time.

### 2.8. In Vitro Antifungal Activity of Hexaconazole-Micelles Nanodelivery System

**Figure 8:**
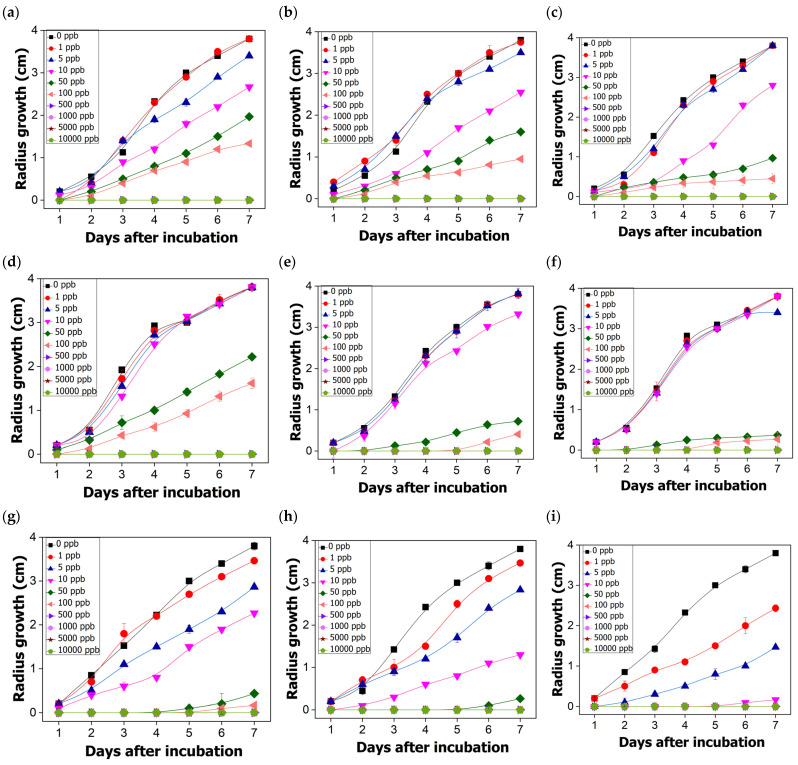
Growth curves of *G. boninense* treated for 7 days with HMBS-1 (**a**), HMBS-2 (**b**), HMBS-4 (**c**), HMDS-1 (**d**), HMDS-2 (**e**), HMDS-4 (**f**) and HMT-6 (**g**), HMT-8 (**h**) and HMT-10 (**i**).

**Figure 9:**
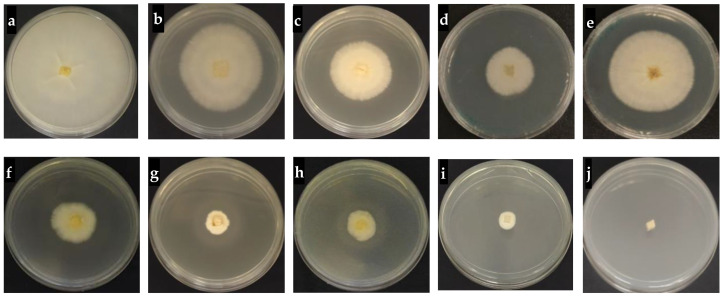
The inhibition zone of *Ganoderma boninense* incubated with 50 ppb of the sample after 7 days; control (**a**) HMBS-1 (**b**), HMBS-2 (**c**), HMBS-4 (**d**), HMDS-1 (**e**), HMDS-2 (**f**), HMDS-4 (**g**) and HMT-6 (**h**), HMT-8 (**i**) and HMT-10 (**j**).

The fungicidal activity of hexaconazole-micelles nanodelivery systems against *Ganoderma boninense* was tested with range concentrations of 1–10,000 ppb and the growth pattern is displayed in Figure 8. In general, the growth curve has shown that *G. boninense* was still growing at a concentration of 50 ppb when treated with HMBS and HMDS, compared to the zero growth for HMT sample. The inhibition zone of the Ganoderma pathogen by each surfactant type of the hexaconazole-micelles nanodelivery system is shown in Figure 9.

**Figure 10:**
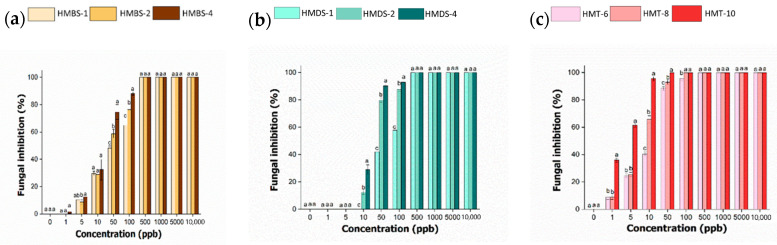
The percentage of inhibition radical growth (PIRG) against concentration (ppb) of hexaconazole-micelles nanodelivery systems based on different surfactants, HMBS (**a**), HMDS (**b**) and HMT (**c**) at day 7. Note: means that do not share a letter are significantly different, where *p* < 0.05 is considered a significant value.

To study the effect of surfactant concentration on the pathogen inhibition, the percentage of inhibition for all the hexaconazole-micelle nanodelivery systems were recorded on day 7 and the results were analyzed. Each concentration of different surfactants used to produce the hexaconazole-micelle nanodelivery systems was used in this study and the significant difference among three samples in each surfactant system are compared, as shown in Figure 10.

The hexaconazole-micelle produced based on sodium dodecylbenzene sulfonate (HMBS) (Figure 10A), the inhibition starts to occur at 1 ppb. However, in the range of 1–10 ppb, not much difference inhibition can be observed between the samples. It was observed that the mean and standard deviation of each sample, HMBS-1, HMBS-2 and HMBS-4, are showing significance at a concentration of 50 and 100 ppb. The plot also indicates that the sample prepared with a higher percentage of SDBS, HMBS-4 had significant inhibition of 74–88%, followed by HMBS-2 and HMBS-1 with a value of 58–76% and 48–64%, respectively. Starting at 500 ppb and above, there is no significant difference for all three samples, as they have fully inhibited the *G. boninense* fungi growth.

There is no obvious inhibition at low concentration for micelle-based sodium dodecyl sulfate (HMDS) (Figure 10B) that can be observed. The micelle-SDS samples showing rapid inhibition, starting at 10 ppb to 50 ppb, and significant differences were observed. The percentage inhibition for HMDS-1 increases from 29 to 90%, followed by HMDS-2 with 12 to 79% and the lowest is the SDS concentration, HMDS-1 with value 0 to 42%. The mean inhibition for each sample does not show a significant effect when up to 500 ppb were used, similar to the HMBS system.

Figure 10C represents the HMT system, where the inhibition of the samples towards *Ganoderma* starts as earlier as at 1 ppb. The sample with the highest percentage of T80, i.e., HMT-10 has shown a triple inhibition value of 32% compared to the lower surfactant concentration. It was observed that there is no significant inhibition between HMT-6 and HMT-8, for the concentration up to 5 ppb. At 10 ppb, the HMT-10 shows significant inhibition with about 95%, which is almost double the value of that of HMT-6. The inhibition continues and the *G. boninense* was fully inhibited at 100 ppb by both HMT-8 and HMT-10.

Table 2 summarizes overall EC_50_ values for all the hexaconazole micelles nanodelivery systems based on different surfactants. As shown in the table, increasing the surfactant concentration resulting in a lower EC_50_ value. For all the three types of micelles, the EC_50_ value for HMT was found to be the lowest with a value of 2–13, but the surfactant amount used was the highest, 6–8%, compared to the other systems. The smaller particles of HMT are presumably the reason for this.

**Figure 11:**
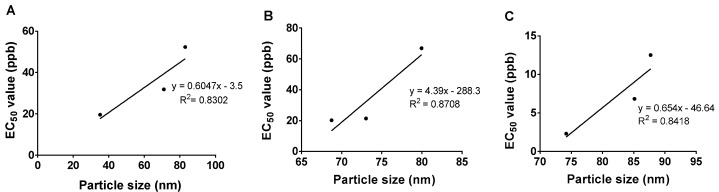
The relationship between the particle size and the EC_50_ values of the as-synthesized hexaconazole-micelle nanodelivery systems: (**A**) HMBS; (**B**) HMDS; and (**C**) HMT towards *G. boninense*.

The correlation of micelle size with the *Ganoderma* inhibition has been studied and the results are summarized in Figure 11. For all the systems, the plot shows almost a linear relationship between particle size and EC_50_ value, where the latter increased when the particle size was increased. This finding suggests that the inhibition efficiency towards *G. boninense* growth was higher when the particle size is reduced. This is due to the higher surface area when the particle size becomes smaller, indicating that the nano micelles’ formulation was capable of diffusing into the fungal cell membrane more effectively, disrupting the cell and then causing cell death [23].

Table 3 demonstrates the interaction between the concentration and surfactant towards the *Ganoderma boninense* growth under the two-way ANOVA. Table 3 indicates that concentration and type of surfactants affect fungal growth. By comparing both factors, the concentration applied has a highly significant effect on the fungal growth, because the *F*-value for the concentration is higher than the surfactants.

By comparing the surfactant types used for the formulation, it can be concluded that Tween 80 gives the highest significant effect to the growth, followed by SDS, and SDBS is the lowest. This finding can be associated with the presence of polyoxyethylene units in the Tween 80 surfactant. This polyoxyethylene group reduced the hydrophobic character of surfactant and thus increases the adsorption efficacy on hydrophobic surfaces such as hexaconazole [20].

This occurrence is also supported by the obtained EC_50_ values as mentioned earlier in Table 2. The efficient adsorption of Tween 80 in its micelle nanodelivery system, HMT has lowered the EC_50_ value, showing better micelle inhibition towards the pathogen. However, the most significant interaction occurred between hexaconazole and SDS, with a value of 377 while the least interaction was observed between hexaconazole and SDBS, with a value of only 18.

## 3. Materials and Methods

### 3.1. Chemical Reagents

Hexaconazole (C_14_H_17_Cl_2_N_3_O), purity (95%) was purchased from Guangzhou, China. Sodium dodecylbenzene sulfonate (MF = C_18_H_29_NaO_3_S, MW = 342.4, 99%), sodium dodecyl sulfate (MF = NaC_12_H_25_SO_4,_ MW = 288.372, 98%), Tween 80 (MF = C_64_H_124_O_26,_ MW = 1310) and all other reagents were obtained from Sigma Aldrich and used as received without any further purification. Deionized water was used during this study.

### 3.2. Micelle Preparation

An aqueous solution was prepared by dissolving 1.226 g of sodium dodecylbenzene sulfonate (SDBS) or sodium dodecyl sulfate (SDS) or Tween 80 (T80) into 150 mL deionized water. Then, 100 mL acetone containing 6.614 g hexaconazole was introduced into the aqueous solution prepared above while stirring using a magnetic stirrer at 40–45 °C until complete evaporation. The micelle solution was placed in an oil bath shaker for about 16 h at a temperature of 70 °C. The sample was then centrifuged and subsequently dried in an oven at 40 °C. The as-synthesized samples were labeled as hexaconazole–based SDBS (HMBS), hexaconazole-based SDS (HMDS), hexaconazole-based T80 (HMT) micelles, respectively. For the physical mixture, hexaconazole and surfactants were mixed thoroughly in a mortar and pestle. The ratio of fungicide and surfactant prepared for the study was the same as for the micelles prepared above.

### 3.3. Measurement of Critical Micelle Concentration

The critical micelle concentration (CMC) of the micelle was determined using the pyrene fluorescence probe spectrometry [24]. In this technique, the solutions with different concentrations of hexaconazole micelles in the range of 1 × 10^−2^–4.0% were prepared in 10 mL deionized water. The mixtures were shaken until homogenous samples were produced. 1.0 mL of pyrene solution in methanol (0.3 g/L) was added to each vial. The mixtures were then shaken again to maximally dissolve pyrene in the prepared solution. After sonication for 30 min, the samples were incubated at room temperature for approximately 17 h. The supernatant solution was collected and analyzed using a UV-Visible spectrophotometer at 230 and 319 nm. The absorbance difference of (λ_230_ − λ_319_) was plotted against the log concentration of micelles. The CMC of hexaconazole micelle was determined by taking the micelle concentration where the intersection point of two lines occurs.

### 3.4. Characterization of Hexaconazole Micelles

X-ray Diffraction (XRD) analysis was carried out to determine the crystallinity of hexaconazole in the micelle system using a Shimadzu diffractometer, operating with the voltage of 40 kV and the current of 25 mA. Data were collected in the 2θ range of 5–50 degrees at a scan rate of 4.0 deg/min. The composition of the synthesized micelle was also confirmed by the Fourier-transform infrared (FTIR) spectra using a PerkinElmer 1725X spectrophotometer by the ATR technique with a wavenumber of 400–4000 cm^−1^. The thermal properties were studied using a Mettler Toledo instrument. The samples were placed in an aluminum pan and heated in the range of 25–1000 °C with 50 mL/min nitrogen flow at a rate of 10 °C/min. Field emission scanning electron microscope (FESEM), JEOL JSM-6400 was used to study the shape and morphology of the micelle.

### 3.5. Measurement of Fungicide Loading in Micelle System

The loading of hexaconazole in the micelle system was conducted by diluting 10 mg of micelle into 1 mL of methanol as was described previously [25]. The hexaconazole content was measured using ultraviolet-visible spectrophotometry (UV-Vis) at a wavelength of 227 nm. The hexaconazole concentration is calculated based on the absorbance value recorded using a calibration curve. The fungicide loading was quantified using Equation (1) [26].

(1) $$\mathrm{Fungicide}\;\mathrm{loading}\;\mathrm{content}\;(\%)\;=\;\frac{\mathrm{Weight}\;\mathrm{of}\;\mathrm{hexaconazole}\;\mathrm{in}\;\mathrm{micelle}\;}{\mathrm{Weight}\;\mathrm{of}\;\mathrm{micelle}}\;\times \;100$$

(2) $$\mathrm{Encapsulation}\;\mathrm{efficiency}\;(\%)\;=\;\frac{\mathrm{Experimental}\;\mathrm{hexaconazole}\;\mathrm{loaded}\;\mathrm{micelle}\;}{\mathrm{Actual}\;\mathrm{feeding}\;\mathrm{hexaconazole}\;\mathrm{of}\;\mathrm{micelle}}\;\times \;100$$

### 3.6. Determination of Particle Size and Stability

The average particle size values were evaluated using a Zeta sizer Nano instrument (Malvern Instruments, Malvern, UK). Oil has been used as a dispersion solvent during the size determination. The temperature was fixed at 25 °C for all the measurements and the results were based on triplicate. The stability of the micelle was checked by tracing the changes in particle size distribution during storage at room temperature and after 6 months of storage.

### 3.7. Fungicidal Activity

#### 3.7.1. In Vitro Antifungal Assay

The pure culture of *Ganoderma boninense* was maintained on potato dextrose agar (PDA) at 28 ± 2 °C. For the antifungal assay, Food Poison Technique was used. The Petri plates were prepared by impregnating desired concentration of micelle solution ranging from 0 to 10,000 ppb into sterilized PDA. After solidification of the media, they were inoculated with a 5 mm inoculum plug that was punched aseptically with a sterile cork borer from the seven-day-old culture of fungus. The plates then were sealed and incubated at a temperature of 28 ± 2 °C for 7 days. The blank agar plates with only *Ganoderma boninense* on it served as a control. After incubation time, the radial diameter of the fungus growth was measured in mm. The percentage inhibition of radical growth, PIRG, was evaluated by comparing the colony radius of the poisoned plate (presence of micelle, G_o_) and non-poisoned plate (control, G_c_) as given in Equation (3) [27],

Percentage of inhibition = (G_c_ − G_o_)/G_c_ × 100 (3)

#### 3.7.2. Statistical Analysis

The comparison of inhibition value was statistically analyzed using the one-way analysis of variance (ANOVA) GraphPad Prism Version 6 software (California, USA). Significant differences between samples were determined by Tukey’s Test and a value of *p* < 0.05 was set as significant when comparing to relevant controls. The results are presented as mean ± SD for three independent tests.

## 4. Conclusions

The method used for the preparation of micelle-based agronanofungicide in this work was found to be more easily compared to other previously reported micelle formulations. The use of surfactants enhanced the hexaconazole micelles’ performance by improving their solubility and bioavailability. Due to the improved properties, the amount of the active ingredient (fungicide) needed can be reduced, but with the maximal absorption of the fungicide to the infection site. The systems can be easily dissolved with water before they are applied in fields or plantations using the trunk injection or spraying procedure. This study also indicates that the surfactant concentration can be used to tune the micelle particle size and its stability which subsequently can be used to tailor to their specific application. The present study shows that the use of different surfactants resulted in different antifungal effects against *Ganoderma boninense*. With the tunable particle size at nanometer regime, and therefore higher efficient surface area for adsorption, the hexaconazole micelles-based formulation is a promising candidate to be used as agronanochemical, especially in battling the basal stem rot disease in oil palm.

## Figures and Tables

**Table 1:** Particle size distribution (modes) of the hexaconazole-micelle nanodelivery systems using various surfactant concentrations.

Surfactant Used	Surfactant Concentration (% *w*/*v*)	Formulation Label	PSD * Intensity (nm)	
			Initial	after 6 Months of Storage
Sodium dodecyl benzene sulfonate	1	HMBS-1	83	133
	2	HMBS-2	71	118
	4	HMBS-4	35	90
Sodium dodecyl sulfate	1	HMDS-1	80	150
	2	HMDS-2	73	137
	4	HMDS-4	69	103
Tween 80	6	HMT-6	88	137
	8	HMT-8	87	122
	10	HMT-10	74	91

* PSD = Particle Size Distribution.

**Table 2:** The half-maximal effective concentration, EC_50_ values for hexaconazole-micelle nanodelivery systems prepared at different concentrations and different surfactant types against *G. boninense* after 7 days incubation.

Samples	EC_50_ (ppb)	EC_50_ 95% Confidence Interval	
		Lower Limit	Upper Limit
HMBS-1	52	40	68
HMBS-2	32	26	40
HMBS-4	20	16	25
HMDS-1	67	55	81
HMDS-2	21	16	28
HMDS-4	20	13	31
HMT-6	13	10	15
HMT-8	7	5	9
HMT-10	2	1	4

**Table 3:** Interaction of the sample concentration and the surfactant towards the *Ganoderma boninense* growth.

Growth	Micelle	Concentration	Surfactant	Concentration × Surfactant
*p*-value	HMBS	0	0	0
	HMDS	0	0	0
	HMT	0	0	0
*F*-value	HMBS	3909	55	18
	HMDS	25,348	1291	377
	HMT	12,690	1368	309

## Data Availability

Not applicable.

## References

[1] Idris D., Arifin S., Ahmad H. (2004). Prolonging the productive life of Ganoderma-infected palms with hexaconazole. MPOB Inf. Ser..

[2] Maznah Z., Halimah M., Ismail S., Idris A.S. (2015). Dissipation of the fungicide hexaconazole in oil palm plantation. Environ. Sci. Pollut. Res..

[3] Baird T., DeLorenzo M.E. (2009). Descriptive and mechanistic toxicity of conazole fungicides using the model test algaDunaliella tertiolecta(chlorophyceae). Environ. Toxicol..

[4] Muhamad H., Zainol M., Sahid I., Abu Seman I. (2012). Determination of hexaconazole in field samples of an oil palm plantation. Drug Test. Anal..

[5] Mustafa I.F., Hussein M.Z., Saifullah B., Idris A.S., Hilmi N.H.Z., Fakurazi S. (2018). Synthesis of (Hexaconazole-Zinc/Aluminum-Layered Double Hydroxide Nanocomposite) Fungicide Nanodelivery System for Controlling Ganoderma Disease in Oil Palm. J. Agric. Food Chem..

[6] Maluin F.N., Hussein M.Z., Yusof N.A., Fakurazi S., Idris A.S., Hilmi N.H.Z., Daim L.D.J. (2019). Preparation of Chitosan-Hexaconazole Nanoparticles as Fungicide Nanodelivery System for Combating Ganoderma Disease in Oil Palm. Molecules.

[7] Fait M.E., Bakas L., Garrote G.L., Morcelle S.R., Saparrat M.C.N. (2019). Cationic surfactants as antifungal agents. Appl. Microbiol. Biotechnol..

[8] Wu D., Lu J., Zhong S., Schwarz P., Chen B., Rao J. (2019). Influence of nonionic and ionic surfactants on the antifungal and mycotoxin inhibitory efficacy of cinnamon oil nanoemulsions. Food Funct..

[9] Jibrin M.O., Liu Q., Jones J.B., Zhang S. (2021). Surfactants in plant disease management: A brief review and case studies. Plant Pathol..

[10] Miyagishi S. (1976). The Effect of Organic Additives on the Thermodynamic Parameters of Micellization. Bull. Chem. Soc. Jpn..

[11] Liang N., Sun S., Gong X., Li Q., Yan P., Cui F. (2018). Polymeric Micelles Based on Modified Glycol Chitosan for Paclitaxel Delivery: Preparation, Characterization and Evaluation. Int. J. Mol. Sci..

[12] Huang P., Liu A., Kang L., Zhu M., Dai B. (2018). Heteropoly acid supported on sodium dodecyl benzene sulfonate modified layered double hydroxides as catalysts for oxidative desulfurization. New J. Chem..

[13] Singh M.K., Agarwal A., Gopal R., Swarnkar R.K., Kotnala R.K. (2011). Dumbbell shaped nickel nanocrystals synthesized by a laser induced fragmentation method. J. Mater. Chem..

[14] Choudhury S.R., Mandal A., Chakravorty D., Gopal M., Goswami A. (2013). Evaluation of physicochemical properties, and antimicrobial efficacy of monoclinic sulfur-nanocolloid. J. Nanopart. Res..

[15] King S., McCafferty L., Stolojan V., Silva S.R.P. (2015). Highly aligned arrays of super resilient carbon nanotubes by steam purification. Carbon.

[16] Ahmed A.A.A., Talib Z.A., Hussein M.Z. (2015). Influence of sodium dodecyl sulfate concentration on the photocatalytic activity and dielectric properties of intercalated sodium dodecyl sulfate into Zn–Cd–Al layered double hydroxide. Mater. Res. Bull..

[17] Ramimoghadam D., Bin Hussein M.Z., Taufiq-Yap Y.H. (2012). The Effect of Sodium Dodecyl Sulfate (SDS) and Cetyltrimethylammonium Bromide (CTAB) on the Properties of ZnO Synthesized by Hydrothermal Method. Int. J. Mol. Sci..

[18] Larson N.R., Wei Y., Prajapati I., Chakraborty A., Peters B., Kalonia C., Hudak S., Choudhary S., Esfandiary R., Dhar P. (2020). Comparison of Polysorbate 80 Hydrolysis and Oxidation on the Aggregation of a Monoclonal Antibody. J. Pharm. Sci..

[19] Mukwada L.T., Mochane M.J., Motaung T.E., Motloung S.V., Koao L.F. (2017). Effect of sodium dodecylbenzene sulphonate modifier and PP-g-MA on the morphology and thermal conductivity of PP/EG composites. Plast. Rubber Compos..

[20] Parhizkar M., Edirisinghe M.J., Stride E. (2014). The effect of surfactant type and concentration on the size and stability of microbubbles produced in a capillary embedded T-junction device. RSC Adv..

[21] Landfester K., Bechthold N., Tiarks A.F., Antonietti M. (1999). Formulation and Stability Mechanisms of Polymerizable Miniemulsions. Macromolecules.

[22] Hecht L.L., Wagner C., Landfester K., Schuchmann H.P. (2011). Surfactant Concentration Regime in Miniemulsion Polymerization for the Formation of MMA Nanodroplets by High-Pressure Homogenization. Langmuir.

[23] Mustafa I.F., Hussein M.Z. (2020). Synthesis and Technology of Nanoemulsion-Based Pesticide Formulation. Macromolecules.

[24] Fu J., Cai Z., Gong Y., O’Reilly S., Hao X., Zhao D. (2015). A new technique for determining critical micelle concentrations of surfactants and oil dispersants via UV absorbance of pyrene. Colloids Surf. A Physicochem. Eng. Asp..

[25] Li F., Danquah M., Mahato R.I. (2010). Synthesis and Characterization of Amphiphilic Lipopolymers for Micellar Drug Delivery. Biomacromolecules.

[26] Ali M.M., Sadeghizadeh M., Najafi F., Ardestani S.K., Erfani-Moghadam V., Khaniki M., Rezaei A., Zamani M., Khodayari S., Khodayari H. (2015). Encapsulation of Curcumin in Diblock Copolymer Micelles for Cancer Therapy. BioMed Res. Int..

[27] Skidmore A., Dickinson C. (1976). Colony interactions and hyphal interference between Septoria nodorum and phylloplane fungi. Trans. Br. Mycol. Soc..

